# Perceptions of Aging and Inflammation in the Context of Older Adult Married Couples

**DOI:** 10.1093/geroni/igaa057.1855

**Published:** 2020-12-16

**Authors:** Ashley Ermer, Dikla Segel-Karpas

**Affiliations:** 1 Montclair State University, Montclair, New Jersey, United States; 2 University of Haifa, Haifa, Hefa, Israel

## Abstract

The current study takes a dyadic perspective to understand how self-perceptions of aging are associated with C-reactive protein, an inflammation marker, among older adult married couples. The potential moderating role of marital support and strain are also examined. Respondents include 668 married couples who participated in the 2014 wave of the Health and Retirement Study. Actor-Partner Interdependence Models were conducted in Mplus. Age, functional limitations, income, and race served as covariates. Husbands’ greater positive perceptions of aging were significantly associated with their own lower levels of inflammation. Husbands’ greater positive perceptions of aging were significantly associated with lower levels of inflammation for women who reported lower levels of marital strain; this was not the case for women who reported higher levels of marital negativity. This study exemplifies how relationship factors are necessary to consider when examining age perceptions and health among marrieds.

